# Reliable Identification of Environmental *Pseudomonas* Isolates Using the *rpoD* Gene

**DOI:** 10.3390/microorganisms8081166

**Published:** 2020-07-31

**Authors:** Léa Girard, Cédric Lood, Hassan Rokni-Zadeh, Vera van Noort, Rob Lavigne, René De Mot

**Affiliations:** 1 Centre of Microbial and Plant Genetics, KU Leuven, Kasteelpark Arenberg 20, 3001 Leuven, Belgium; lea.girard@kuleuven.be (L.G.); cedric.lood@kuleuven.be (C.L.); vera.vannoort@kuleuven.be (V.v.N.); 2 Department of Biosystems, Laboratory of Gene Technology, KU Leuven, Kasteelpark Arenberg 21, 3001 Leuven, Belgium; rob.lavigne@kuleuven.be; 3 Zanjan Pharmaceutical Biotechnology Research Center, Zanjan University of Medical Sciences, 45139-56184 Zanjan, Iran; hassan.roknizadeh@gmail.com; 4 Institute of Biology, Leiden University, Sylviusweg 72, 2333 Leiden, The Netherlands

**Keywords:** species identification, taxonomy, phylogeny, diversity, genome sequencing

## Abstract

The taxonomic affiliation of *Pseudomonas* isolates is currently assessed by using the 16S rRNA gene, MultiLocus Sequence Analysis (MLSA), or whole genome sequencing. Therefore, microbiologists are facing an arduous choice, either using the universal marker, knowing that these affiliations could be inaccurate, or engaging in more laborious and costly approaches. The *rpoD* gene, like the 16S rRNA gene, is included in most MLSA procedures and has already been suggested for the rapid identification of certain groups of *Pseudomonas.* However, a comprehensive overview of the *rpoD*-based phylogenetic relationships within the *Pseudomonas* genus is lacking. In this study, we present the *rpoD*-based phylogeny of 217 type strains of *Pseudomonas* and defined a cutoff value of 98% nucleotide identity to differentiate strains at the species level. To validate this approach, we sequenced the *rpoD* of 145 environmental isolates and complemented this analysis with whole genome sequencing. The *rpoD* sequence allowed us to accurately assign *Pseudomonas* isolates to 20 known species and represents an excellent first diagnostic tool to identify new *Pseudomonas* species. Finally, *rpoD* amplicon sequencing appears as a reliable and low-cost alternative, particularly in the case of large environmental studies with hundreds or thousands of isolates.

## 1. Introduction

*Pseudomonas* species are ubiquitous bacteria present in terrestrial, aquatic, and marine environments [1,2]. While several species are a threat to human health or food industry, with detrimental impacts on crops and aquaculture, the large majority of the species are commensals [3,4,5]. In fact, several species have been described as potential biocontrol agents to fight against diverse plant pathogens but also for bioaccumulation or biodegradation of pollutants [6,7]. Furthermore, *Pseudomonas* species are known producers of a wide diversity of bioactive secondary metabolites with potentially high added value [8]. Consequently, it is of great interest to be able to identify *Pseudomonas* isolates, in a fast and inexpensive way, to monitor their occurrence and diversity in the environment.

To date, the identification of bacteria in microbiology is still based on the 16S rRNA gene sequence, although its discriminative power is often limited to the delineation of groups or clades within a particular genus [9]. For *Pseudomonas*, the 16S rRNA gene allows the differentiation with sister genera in the *Pseudomonadaceae* family (*Cellvibrio*, *Oblitimonas*, *Thiopseudomonas*, and *Ventosimonas*) and the delineation of the three main lineages (*P. aeruginosa*, *P. fluorescens,* and *P. pertucinogena*) [10,11,12]. However, intra-genomic heterogeneities originate from multiple copies of the 16S rRNA gene in *Pseudomonas* genomes, and, based on previously established cutoff values (between 98.2 to 99% of similarity), it is not possible to differentiate environmental isolates at the species level [9,12,13,14]. Therefore, MultiLocus Sequence Analysis (MLSA), based on the concatenation of four housekeeping genes (16S rRNA, *gyrB*, *rpoB,* and *rpoD*), has been proposed for the identification of *Pseudomonas* isolates [15,16]. Nevertheless, the expansion of genomics in bacterial taxonomy, and attractive prices, have led to the genome sequencing of many environmental isolates of *Pseudomonas* [17,18]. Recently, the *Pseudomonas* phylogeny based on the 16S rRNA gene and the concatenation of 4, 100, and 120 genes was compared to whole genome analyses [12]. As a result, it seems that the MLSA based on four housekeeping genes has the best price–performance ratio, although the use of only two housekeeping genes (*rpoB* and *rpoD*) can also be found in the literature [19,20]. However, these methodologies are time-consuming, demanding, and/or expensive, particularly in the case of large environmental studies with hundreds or thousands of isolates. Consequently, the use of the 16S rRNA gene to identify *Pseudomonas* isolates remains relevant despite being prone to misidentifications, as observed frequently in public databases [21,22].

The *rpoD* gene is included in most MLSA procedures and was previously proposed to identify *Pseudomonas* species in environmental samples [23,24] or for the rapid identification of isolates belonging to the *Pseudomonas syringae* complex [25]. However, since these studies included only a limited number of *Pseudomonas* species, a genus-wide comprehensive overview of the *rpoD* phylogeny is presently missing. In this study, we present the *rpoD*-based *Pseudomonas* phylogeny, including a total of 217 type strains for which genomes were available in public databases. Secondly, we adapted the methodology described by Mulet et al. (2011) to sequence the *rpoD* gene and identify 145 environmental *Pseudomonas* isolates. To test our *rpoD*-based taxonomic affiliations, we sequenced one-third of these isolates with Illumina and used whole genome analysis (Average Nucleotide Identity and digital DNA–DNA hybridization) for comparison with the established taxonomy. We show here that the *rpoD* locus streamlines the identification of *Pseudomonas* isolates from a labor and cost perspective and provides, in comparison with the 16 rRNA gene, an excellent tool to accurately affiliate isolates.

## 2. Materials and Methods

All type strains used in this study, together with NCBI accession numbers, are listed in Table S1. The environmental isolates of *Pseudomonas*, their origin, and their NCBI accession number (*rpoD* and/or genome) are listed in Table S2.

**Figure 1:**
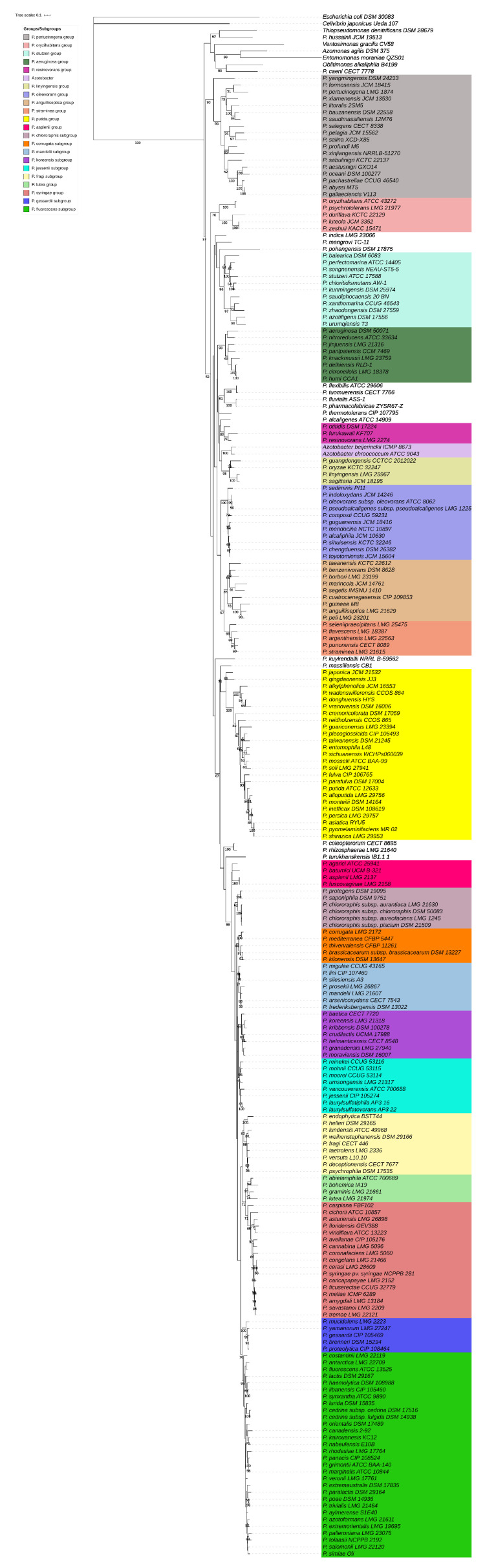
Maximum likelihood phylogenetic tree based on a ~650 bp fragment of the *rpoD* gene for 226 type strains (217 *Pseudomonas*, six from sister genera, and two *Azotobacter*). The tree was constructed using the GTR+G+I model (MEGA-X), and only bootstrap values higher than 50% are indicated. The *rpoD* sequence of *Escherichia coli* was included as an outgroup.

**Figure 2:**
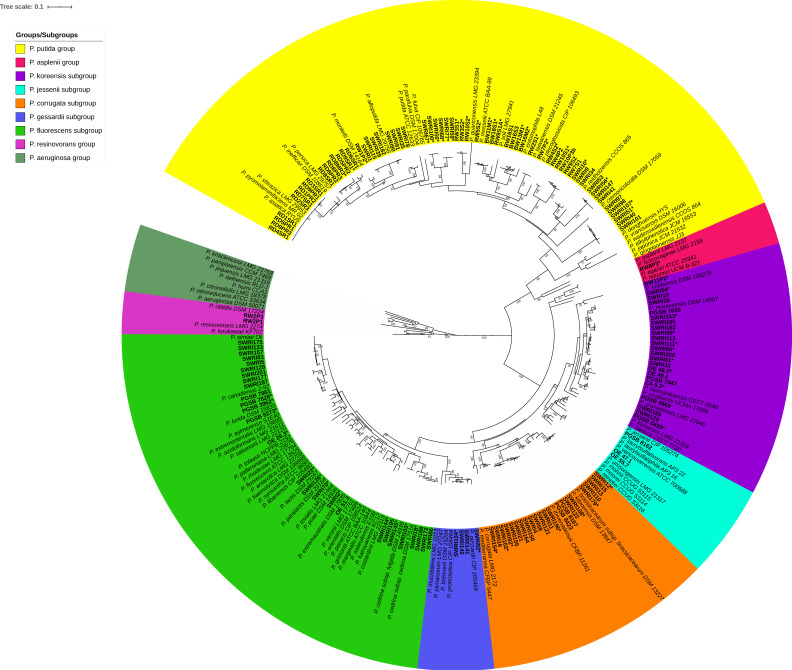
Maximum likelihood phylogenetic tree based on a ~650 bp fragment of the *rpoD* gene, including the 145 environmental isolates and related groups/subgroups. The tree was constructed using the GTR+G+I model (MEGA-X), and only bootstrap values higher than 50% are indicated. The *P. aeruginosa* group was used as the outgroup. Environmental isolates are highlighted in bold. * Indicates genome sequenced isolates.

To avoid DNA extractions, PCR was performed on cell lysates. One colony of each strain was suspended in 50 µL of Milli-Q water in 96-well PCR microplates, and plates were immersed three times, alternating between liquid nitrogen and water bath (+70 °C). The lysates were then stored at −20 °C until their use as PCR templates. PCR amplifications of the *rpoD* gene were performed using previously designed primers (PsEG30F and PsEG790R; [23]) and KAPA2G Fast HotStart ReadyMix (Sigma–Aldrich, Saint-Louis, Missouri, USA). Cycling conditions were as follows: initial denaturation at 95 °C for 5 min followed by 30 cycles of annealing at 60 °C for 30 s, extension at 72 °C for 30 s and denaturation at 95 °C for 15 s, and reactions were completed at 72 °C for 2 min. PCR products were then purified using the GenElute PCR Clean-Up kit (Sigma–Aldrich Saint-Louis, Missouri, USA). Sequencing was performed using the reverse primer PsEG790R first, and, in case of failed sequencing, the forward primer PsEG30F was used. Purified PCR products were then sequenced using Sanger sequencing (Macrogen Europe, Amsterdam, The Netherlands) to obtain a final fragment of approximately 650 bp. The *rpoD* sequences of 145 environmental isolates were aligned to those from the 217 type strains of *Pseudomonas* to generate a similarity matrix based on a ~650 bp fragment (Table S3). A first phylogenetic tree containing all type strains of *Pseudomonas* and members of the sister genera was constructed to confirm the relatedness between validly published species (Figure 1; Table S1). A second, restricted tree shows the phylogenetic relationship of our 145 *Pseudomonas* isolates with the known *Pseudomonas* species (Figure 2; Table S2). MEGA-X was used to estimate the best evolutionary model. Both trees were constructed using the general time-reversible model (GTR+G+I), and bootstrap values were calculated based on 1000 replications [26]. iTOL was then used to annotate the trees and create high-quality figures [27].

To validate the *rpoD*-based taxonomic affiliation, the whole genomes of 55 environmental isolates were sequenced. Three strains, namely SWRI103, BW11P2, and BW11M1, were included in this analysis but were previously sequenced by our group [28,29,30]. Briefly, genomic DNA was extracted using the Gentra Puregene Yeast/Bact. Kit (Qiagen, Hilden, Germany). A first subset of the genomes was sequenced by BASECLEAR (Leiden, The Netherlands) using the Nextera XT library preparation kit and the Illumina MiSeq sequencer. The second batch of strains was sequenced in-house using the Nextera Flex preparation kit and the Illumina MiniSeq device. All libraries were sequenced using a paired-end approach (2 × 150 bp), and the genome coverage was routinely above 40×. The quality of the Illumina reads was assessed using FastQC v. 0.11.9 and Trimmomatic v. 0.38 for adapter clipping, quality trimming (LEADING:3 TRAILING:3 SLIDINGWINDOW:4.15), and minimum length exclusion (>50 bp) [31]. De novo genome assembly was performed with the SPAdes assembler v. 3.13.0 [32].

Bioinformatics calculations, such as the Average Nucleotide Identity (ANI) and digital DNA–DNA Hybridization (dDDH), are now commonly used to describe new species or to affiliate strains to a specific taxon [20,33]. Threshold values for species delineation are, respectively, between 95 and 96.5 for ANI and 70 for dDDH [13,34,35,36,37]. In this study, we calculated ANIb values between a total of 275 *Pseudomonas* strains (217 type strains and 58 environmental strains) using pyani v0.2.10 with default parameters and the ANIb method. ANIb values in Table 1, Table 2 and Table 3 are the averages of the bidirectional comparisons. In the case of ambiguous ANIb values (between 95 and 96.5%), we supplemented the analysis with the calculation of dDDH using the online tool Genome-to-Genome Distance Calculator GGDC (https://ggdc.dsmz.de/home.php, June 2020) [34,35]. Finally, in order to evaluate reliability of *rpoD*-based taxonomic affiliations, we calculated the correlation *rpoD*/ANIb using the statistical functions included in the SciPy Python package [38].

## 3. Results and Discussion

### 3.1. rpoD Phylogeny

The *rpoD* phylogeny is based on 217 type strains of *Pseudomonas*, six representative strains from sister genera (*Cellvibrio*, *Oblitimonas*, *Thiopseudomonas*, and *Ventosimonas*), and two *Azotobacter* type strains (Figure 1, Table S3). The *rpoD* allows the differentiation from members of sister genera (nucleotide identities ranging from 42.51 to 65.68%) and the discrimination of the three *Pseudomonas* lineages (*P. pertucinogena*, *P. aeruginosa,* and *P. fluorescens*) (Figure 1 and Table S3). Within the three lineages, the *rpoD*-based grouping of type strains accurately reflects previously defined groups and subgroups, based on the concatenation of 4 or 100 genes [12,16,37]. The *P. aeruginosa* lineage is divided in eight phylogenetic groups (*P. oryzihabitans*, *P. stutzeri*, *P. oleovorans*, *P. aeruginosa*, *P. resinovorans*, *P. linyingensis*, *P. anguilliseptica,* and *P. straminea*). The *P. fluorescens* lineage is divided in five phylogenetic groups (*P. putida*, *P. asplenii*, *P. lutea*, *P. syringae,* and *P. fluorescens*), and overall these groups are supported by relatively high bootstrap values (Figure 1 and Figure 2). The genus *Azotobacter* is included in the *P. aeruginosa* lineage with highest similarities with members of the *P. resinovorans* group (74.55 to 76.51%; (Table S3)). The *P. lutea* and *P. syringae* groups are included in the *P. fluorescens* group, and a total of 16 species are scattered across the three (i.e., *P. hussainii*, *P. caeni*, *P. indica*, *P. mangroviangrove*, *P. pohangensis*, *P. flexibilis*, *P. tuomuerensis*, *P. fluvialis*, *P. pharmacofabricae*, *P. thermotholerans*, *P. alcaligenes*, *P. kuykendallii*, *P. massiliensis*, *P. coleopterorum*, *P. rhizosphaerae,* and *P. turukhanskensis*). Conversely, *P. sichuanensis*, *P. guangdongensis,* and *P. cuatrocienegasensis* are integrated, respectively, in the *P. putida*, *P. linyingensis,* and *P. anguilliseptica* groups [12,16,37].

### 3.2. Species Delineation

Within the *Pseudomonas* genus, the lowest nucleotide identity based on the *rpoD* locus among the 217 type strains included in this study was 51.57%. We observed that the lowest intra-group nucleotide identity ranged from 66.06 to 88.54% within, respectively, the *P. pertucinogena* and *P. oleovorans* group. Overall, all type strains were differentiated at a species level with a cutoff value of 98% identity (Table S3), which is supported by ANIb values <95% (Table S4). On the other hand, a certain number of *Pseudomonas* species were already described as synonymous, and we highlighted, in Table 1, ten cases where similarities based on *rpoD* (>98%) and ANIb values (>96.5%) were consistent with previous observations [12,16,33,37].

However, we also observed limitations where similarities based on *rpoD* were not concordant with ANIb values (Table 2). MLSA was described as an efficient tool to identify *Pseudomonas* isolates, and a threshold value of 97% was recently proposed to differentiate strains at the species level [12]. Consequently, we also performed MLSA (following previous instructions [15]) to determine if these misinterpretations could be avoided by the use of multiple loci (Table S5). 

It is important to note that in 12 cases out of 15, MLSA and *rpoD* led to the same misinterpretations, while in three cases, incorrect affiliations were made by using the single locus. Overall, among the 23,436 pairwise comparisons, including the 217 type strains, only in 0.017% of the cases two strains belonging to the same species presented an identity < 98%, and in 0.047% of the cases, two strains belonging to different species presented an identity > 98%. In order to evaluate the overall reliability of these *rpoD*-based affiliations, we calculated the correlation between *rpoD* similarities (Table S3) and ANIb values (Table S4). We included 275 strains (217 type strains and 58 environmental strains) and obtained a Pearson correlation coefficient of 0.872 (*p*-Value < 10e-300).

### 3.3. Identification of Environmental Isolates

A total of 145 environmental *Pseudomonas* isolates were identified using the *rpoD* gene (taxonomic affiliations are summarized in Table S2). Briefly, *Pseudomonas* strains were isolated from banana plants (*n* = 7), rice (*n* = 33), maize (*n* = 16), or wheat (*n* = 89) and from diverse geographical locations in Belgium, Iran, and Sri Lanka [39,40]. Only two isolates clustered in the *P. resinovorans* group within the *P. aeruginosa* lineage while the majority, 143 isolates, were affiliated to the *P. fluorescens* lineage, respectively belonging to the *P. putida* (*n* = 63), *P. asplenii* (*n* = 1), and *P. fluorescens* (*n* = 79) groups (Figure 2). Within the *P. fluorescens* group, strains are distributed in five subgroups, *P. jessenii* (*n* = 3), *P. gessardii* (*n* = 4), *P. corrugata* (*n* = 22), *P. koreensis* (*n* = 23), and *P. fluorescens* (*n* = 27) (Figure 2). As previously observed with type strains of *Pseudomonas*, we identified a cutoff value of 98% nucleotide identity on the *rpoD* gene to differentiate strains at the species level. On this basis, 56 isolates were identified and affiliated to 20 known *Pseudomonas* species, with the most abundant ones being *P. asiatica* (*n* = 9), *P. simiae* (*n* = 8), and *P. alloputida* (*n* = 6), while the remaining 89 isolates were suspected to represent new species (Tables S2 and S3). These taxonomic affiliations were supported by ANIb calculations between our 58 genomes and all known *Pseudomonas* species included in this study (Table 3 and Table S4). Finally, the 89 unaffiliated isolates were confirmed to belong to 31 new *Pseudomonas* species (listed *Pseudomonas* sp. #1 to #31 in Table 3 and Table S2).

The new species are distributed as follows: *P. putida* group (*n* = 13), *P. asplenii* group (*n* = 1), *P. gessardii* subgroup (*n* = 1), *P. koreensis* subgroup (*n* = 7), *P. corrugata* subgroup (*n* = 4), and *P. fluorescens* subgroup (*n* = 5). Their description and an update of the *Pseudomonas* phylogeny will be published elsewhere (Girard et al., in preparation). Overall, ~95% of taxonomic affiliations based on the *rpoD* gene that were confirmed by whole genome comparison were accurate. However, we also observed limitations (~5% of the cases) where the *rpoD* alone would have led to affiliation to known species (i.e., SWRI103, SWRI126, OE 28.3, Table 3), while based on ANIb/dDDH these strains represent new *Pseudomonas* species. These strains are also affiliated to known species when using MLSA (>97%, Table S5). Therefore, based on a total of 37,402 dual comparisons (217 type strains and 58 environmental strains), a threshold of 98% similarity led to 0.048% of misidentifications.

The 16S rRNA gene is still widely used in microbiology to identify bacteria [9]. However, the presence of multiple copies of this gene can lead to different ribotypes for a given *Pseudomonas* strain [14]. On the other hand, the *rpoD* is present in a single copy, offers a higher resolution than the 16S rRNA gene [12], and appears to be as efficient as MLSA to taxonomically assign environmental isolates (this study). Furthermore, the sequencing of four genes for MLSA or the 16S rRNA gene (i.e., sequencing of both ends to have the longest fragment) generates higher costs than *rpoD* (one end only). Consequently, the use of this locus for the identification of large batches of environmental isolates is advantageous in terms of resolution but also from a practical and financial point of view. Moreover, the widespread distribution of the *rpoD* gene in public databases, especially through its use for MLSA, now makes it an interesting target for metagenomics studies heading to evaluate the diversity of *Pseudomonas* within environmental samples [23].

## 4. Conclusions

In this study, we used a ~650 bp *rpoD* locus to describe the phylogenetic structure of the genus *Pseudomonas*. We provide a genus-wide overview of the taxonomy and an integrative study making the link between single locus and whole genome analysis. The use of the *rpoD* allowed us to accurately assign strains to specific phylogenetic groups or subgroups and affiliate strains to known *Pseudomonas* species. Furthermore, it has proven to be efficient as a first diagnostic tool to identify 31 new species. The high correlation between *rpoD* similarities and ANIb values emphasize the high discriminative power of this gene. Importantly, we show that the *rpoD* is a powerful tool for microbiologists, superior to the 16S rRNA gene, for accurate identification of *Pseudomonas* isolates.

## Figures and Tables

**Table 1:** *Pseudomonas* species considered as synonymous (Tables S3 and S4).

*Pseudomonas* Species		*rpoD*	ANIb	Reclassification
*P. gallaeciensis*	*P. abyssi*	99.24	97.56	*P. gallaeciensis*
*P. citronellolis*	*P. humi*	99.38	96.7	*P. citronellolis*
*P. flexibilis*	*P. tuomuerensis*	98.01	98.69	*P. flexibilis*
*P. fluvialis*	*P. pharmacofabricae*	98.44	98.61	*P. fluvialis*
*P. chengduensis*	*P. sihuiensis*	98.79	96.24	*P. chengduensis*
*P. oleovorans* ^a^	*P. pseudoalcaligenes* ^b^	99.85	97.16	*P. oleovorans*
*P. luteola*	*P. zeshuii*	98.92	97.87	*P. luteola*
*P. asiatica*	*P. pyomelaninifaciens*	100	99.03	*P. asiatica*
	*P. shirazica*	99.85	99.17	
*P. amygdali*	*P. ficuserectae*	99.08	97.42	*P. amygdali*
	*P. meliae*	99.23	98.27	
	*P. savastanoi*	99.54	98.75	
*P. asplenii*	*P. fuscovaginae*	99.38	98.23	*P. asplenii*

^a^*P. oleovorans* subsp. *oleovorans*, ^b^ *P. pseudoalcaligenes* subsp. *pseudoalcaligenes.*

**Table 2:** Inconsistent species affiliations based on the comparison between *rpoD*, 4-genes MultiLocus Sequence Analysis (MLSA), and ANIb (Tables S3–S5). Species differentiation based on *rpoD* < 98%, MLSA < 97%, and ANIb < 96.5 were used.

*Pseudomonas* Species		*rpoD*	4-Genes MLSA	ANIb	Reclassification
*P. chengduensis*	*P. toyotomiensis*	98.76	97.74	94.48	*-*
*P. indoloxidans*	*P. oleovorans* ^a^	98.61	97.51	95.78 ^c^	*-*
	*P. pseudoalcaligenes* ^b^	98.45	97.48	95.42 ^c^	*-*
*P. chloritidismutans*	*P. kunmingensis*	94.79	98.24	96.47 ^d^	*P. chloritidismutans*
*P. oryzihabitans*	*P. psychrotolerans*	97.4	98.60	98.22	*P. oryzihabitans*
*P. grimontii*	*P. marginalis*	99.23	98.41	93.46	*-*
	*P. panacis*	98.31	97.78	88.20	*-*
*P. veronii*	*P. panacis*	94.3	95.96	99.95	*P. veronii*
*P. tremae*	*P. coronafaciens*	92.45	93.86	98.73	*P. tremae*
*P. tremae*	*P. amygdali*	99.54	99.20	85.85	*-*
	*P. ficuserectae*	98.92	97.72	85.74	*-*
	*P. meliae*	99.08	98.40	85.99	*-*
	*P. savastanoi*	99.38	97.58	85.91	*-*
*P. libanensis*	*P. synxantha*	98.15	98.49	95.25 ^c^	*-*
*P. guguanensis*	*P. mendocina*	98.14	94.99	89.21	*-*

^a^*P. oleovorans* subsp. *oleovorans*, ^b^ *P. pseudoalcaligenes* subsp. *pseudoalcaligenes,* ^c^ dDDH < 70%, ^d^ dDDH > 70%.

**Table 3:** Confirmation of *rpoD*-based taxonomic affiliations. Strains for which the similarities based on *rpoD* are not concordant with ANIb values are highlighted in bold.

Group/Subgroup	Strain	Closest-Related Strain	*rpoD*	ANIb	Taxonomic Affiliation
*P. putida* group	RD8MR3	RD9SR1	97.84	94.70	*Pseudomonas* sp. #1
	RD9SR1	RD8MR3	97.84	94.70	*Pseudomonas* sp. #2
	RW1P2	*P. monteilii*	98.00	95.77 *	*Pseudomonas* sp. #3
	SWRI67	SWRI68	93.03	<95	*Pseudomonas* sp. #4
	SWRI100	SWRI77			
	SWRI50	SWRI59			
	SWRI68	SWRI67	93.03	<95	*Pseudomonas* sp. #5
	SWRI77	SWRI100			
	SWRI59	SWRI50			
	RW3S1	BW13M1	84.07	85.36	*Pseudomonas* sp. #6
	RW3S2		84.07	85.32	
	RW10S2		83.92	85.44	
	RW5S2	*P. mosselii*	99.85	99.01	*P. mosselii*
	BW11M1		99.69	99.17	
	BW18S1		99.23	97.36	
	RW9S1A	BW18S1	92.94	89.14	*Pseudomonas* sp. #7
	BW13M1	BW18S1	91.06	94.81	*Pseudomonas* sp. #8
	BW16M2		90.91	94.62	
	RW2S1	*P. taiwanensis*	100	99.63	*P. taiwanensis*
	RW7P2		100	99.67	
	RW4S2	*P. plecoglossicida*	85.69	87.10	*Pseudomonas* sp. #9
	RW10S1	RW4S2	89.52	86.57	*Pseudomonas* sp. #10
	SWRI10	*P. reidholzensis*	92.91	86.65	*Pseudomonas* sp. #11
	SWRI56	*P. reidholzensis*	85.56	85.12	*Pseudomonas* sp. #12
	SWRI107	BW13M1	83.82	85.62	*Pseudomonas* sp. #13
	SWRI51		83.82	85.66	
*P. asplenii* group	RW8P3	*P. asplenii*	89.55	88.26	*Pseudomonas* sp. #14
*P. koreensis* subgroup	BW11P2	*P. kribbensis*	91.06	91.61	*Pseudomonas* sp. #15
	SWRI54	*P. moraviensis*	96.28	91.95	*Pseudomonas* sp. #16
	SWRI153	SWRI54	95.05	89	*Pseudomonas* sp. #17
	SWRI99	SWRI54	93.99	88.44	*Pseudomonas* sp. #18
	SWRI111		94.58	88.56	
	SWRI65		94.43	88.57	
	SWRI81		93.99	88.55	
	OE 48.2	*P. crudilactis*	96.74	91.60	*Pseudomonas* sp. #19
	ZA 5.3	*P. crudilactis*	96.89	93.47	*Pseudomonas* sp. #20
	PGSB 8459	*P. koreensis*	94.61	89.11	*Pseudomonas* sp. #21
*P. corrugata* subgroup	SWRI12	*P. thivervalensis*	95.20	92.84	*Pseudomonas* sp. #22
	SWRI179		95.20	92.85	
	SWRI108	*P. kilonensis*	95.41	95.73*	*Pseudomonas* sp. #23
	SWRI196	*P. kilonensis*	92.15	88.02	*Pseudomonas* sp. #24
	SWRI92	*P. kilonensis*	91.69	87.92	*Pseudomonas* sp. #25
	SWRI102		91.69	87.89	
	SWRI154		90.92	87.66	
*P. gessardii* subgroup	SWRI52	*P. gessardii*	96.92	92.50	*Pseudomonas* sp. #26
	SWRI104	*P. proteolytica*	99.38	98.35	*P. proteolytica*
*P. fluorescen*s subgroup	SWRI18	*P. cedrina* subsp. *cedrina*	98.92	96.94	*P. cedrina subsp. cedrina*
	**SWRI103**	*P. cedrina* subsp. *cedrina*	**98.61**	94.19	*Pseudomonas* sp. #27
	SWRI145	*P. cedrina* subsp. *cedrina*	93.07	88.51	*Pseudomonas* sp. #28
	SWRI144		93.07	88.46	
	SWRI2	*P. poae*	99.54	98.85	*P. poae*
	SWRI70	*P. paralactis*	99.23	98.54	*P. paralactis*
	**SWRI126**	*P. lactis*	**98.92**	95.09*	*Pseudomonas* sp. #29
	SWRI22	*P. lactis*	97.99	94.45	*Pseudomonas* sp. #30
	**OE 28.3**	*P. salomonii*	**98.31**	94.10	*Pseudomonas* sp. #31
	PGSB 8273	*P. lurida*	99.85	99.19	*P. lurida*
	PGSB 3962		99.69	99.26	
	PGSB 7828		99.54	99.27	

* dDDH < 70%.

## Supplementary Materials

The following are available online at https://www.mdpi.com/2076-2607/8/8/1166/s1. Table S1: Species type strains used in this study. Table S2: List of environmental strains used in this study. Origin, NCBI accession number for the *rpoD* gene and/or genome, and taxonomic affiliations are specified. Table S3: *rpoD* similarity matrix, including all type and environmental strains. Table S4: ANIb similarity matrix, including all type and strains for which genomes were available. Table S5: MLSA similarity matrix only, including type and environmental strains included in the limitations of *rpoD*-based taxonomic affiliations.
